# Efficacy and safety of Lenzumestrocel (Neuronata-R**®** inj.) in patients with amyotrophic lateral sclerosis (ALSUMMIT study): study protocol for a multicentre, randomized, double-blind, parallel-group, sham procedure-controlled, phase III trial

**DOI:** 10.1186/s13063-022-06327-4

**Published:** 2022-05-18

**Authors:** Jae-Yong Nam, Tae Yong Lee, Kwijoo Kim, Sehwan Chun, Min Sung Kim, Jin-Hong Shin, Jung-Joon Sung, Byoung Joon Kim, Byung-Jo Kim, Ki-Wook Oh, Kyung Suk Kim, Seung Hyun Kim

**Affiliations:** 1 Central Research Center, Corestem Inc, Seoul, South Korea; 2 Department of Neurology, Pusan National University, Yangsan, South Korea; 3 Department of Neurology, Seoul National University, Seoul, South Korea; 4 Department of Neurology, Samsung Medical Center, Seoul, South Korea; 5 Department of Neurology, Korea University Anam Hospital, Seoul, South Korea; 6 College of Pharmacy, Chungbuk National University, Cheongju, South Korea; 7 Department of Neurology, Hanyang University Hospital, Seoul, South Korea

**Keywords:** Lenzumestrocel, Amyotrophic lateral sclerosis, Bone marrow-derived mesenchymal stem cell, Phase III

## Abstract

**Background:**

A single cycle (two repeated treatments) with intrathecal autologous bone marrow-derived mesenchymal stem cells (BM-MSCs, 26-day interval) showed safety and provided therapeutic benefit lasting 6 months in patients with ALS but did not demonstrate long-term efficacy. This phase III clinical trial (ALSUMMIT) protocol was developed to evaluate the long-term efficacy and safety of the combined protocol of single-cycle intrathecal therapy and three additional booster injections of BM-MSC (Lenzumestrocel) treatment in patients with ALS.

**Methods:**

ALSUMMIT is a multicentre, randomized, double-blind, parallel-group, sham procedure-controlled, phase III trial for ALS. The 115 subjects will be randomized (1:2:2) into three groups: (1) study Group 1 (single-cycle, two repeated injections with 26-day interval), (2) study Group 2 (single-cycle + three additional booster injections at 4, 7, and 10 months), and (3) the control group. Participants who have an intermediate rate of disease progression will be included in this trial to reduce clinical heterogeneity. The primary endpoint will be evaluated by combined assessment of function and survival (CAFS), also known as joint rank scores (JRS), at 6 months (study Group 1 vs. control) and 12 months (study Group 2 vs. control) after the first Lenzumestrocel or placebo administration. Safety assessment will be performed throughout the study period. Additionally, after the 56-week main study, a long-term follow-up observational study will be conducted to evaluate the long-term efficacy and safety up to 36 months.

**Discussion:**

Lenzumestrocel is the orphan cell therapy product for ALS conditionally approved by the South Korea Ministry of Food and Drug Safety (MFDS). This ALSUMMIT protocol was developed for the adoption of enrichment enrolment, add-on design, and consideration of ethical issues for the placebo group.

**Trial registration:**

ClinicalTrials.govNCT04745299. Registered on Feb 9, 2021.

Clinical Research Information Service (CRIS) KCT0005954. Registered on Mar 4, 2021.

**Supplementary Information:**

The online version contains supplementary material available at 10.1186/s13063-022-06327-4.

## Administrative information

Note: the numbers in curly brackets in this protocol refer to SPIRIT checklist item numbers. The order of the items has been modified to group similar items (see http://www.equator-network.org/reporting-guidelines/spirit-2013-statement-defining-standard-protocol-items-for-clinical-trials/).

**Table:** 

Title {1}	Efficacy and safety of Lenzumestrocel (Neuronata-R® inj.) in patients with amyotrophic lateral sclerosis (ALSUMMIT study): study protocol for a multi-centre, randomized, double-blind, parallel-group, sham-procedure-controlled, phase III trial
Trial registration {2a and 2b}.	ClinicalTrials.gov Identifier: NCT04745299. Registered on Feb 9 2021 Clinical Research Information Service (CRIS) Identifier: KCT0005954. Registered on Mar 4 2021
Protocol version {3}	Jul 08, 2021 Version 8.0
Funding {4}	Corestem Inc., Manufacturer (Korea)
Author details {5a}	Jae-Yong Nam^1,†^, Tae Yong Lee^1,6,†^, Kwijoo Kim^1^, Sehwan Chun^1^, Min Sung Kim^1,6^, Jin-Hong Shin^2^, Jung-Joon Sung^3^, Byoung Joon Kim^4^, Byung-Jo Kim^5^, Ki-Wook Oh^7^, Kyung Suk Kim^1,*^, Seung Hyun Kim^7,*^ ^1^Central Research Center, Corestem Inc, Seoul, Korea ^2^Department of Neurology, Pusan National University, Yangsan, Korea ^3^Department of Neurology, Seoul National University, Seoul, Korea ^4^Department of Neurology, Samsung Medical Center, Seoul, Korea ^5^Department of Neurology, Korea University Anam Hospital, Seoul, Korea ^6^College of Pharmacy, Chungbuk National University, Cheongju, Korea ^7^Department of Neurology, Hanyang University Hospital, Seoul, Korea
Name and contact information for the trial sponsor {5b}	Kyung Suk Kim, Corestem Inc. Tel: +82-2-497-3711 Post address: 2F, 24, Pangyo-ro 255beon-gil, Bundang-gu, Seongnam-si, Gyeonggi-do 13486 KOREA
Role of sponsor {5c}	Securing funding; delegation of all responsibility regarding the management of trial, data analysis, interpretation, writing reports, management of IDMC; taking part in study design; decision to submit reports for publication.

## Introduction

### Background and rationale {6a}

Amyotrophic lateral sclerosis (ALS), which is known as Lou Gehrig’s disease, is a neurodegenerative disease characterized by progressive loss of selective motor neurons in the brain, brain stem, and spinal cord, which leads to progressive weakness with a fatal outcome due to paralyzed respiratory muscles within a mean of 2–4 years after diagnosis [1, 2]. Riluzole and edaravone are the only approved drugs by the Food and Drug Administration (FDA) with modest treatment effects on ALS progression [3, 4]. While the pathogenic mechanisms of sporadic ALS cases remain unknown, genetic mutations linked to the disease have provided a more accessible target for therapeutic development [5]. The development of therapies that specifically target known ALS mutations, including *chromosome 9 open reading frame 72* (*C9orf72*) and *superoxide dismutase 1* (*SOD1*), *fused in sarcoma* (*FUS*), has been rapidly evolving in recent years [6–8]. Despite the emerging precision of medicine in the genetic cause of ALS, more than 90~95% of patients present with sporadic ALS, and their clinical manifestations are more heterogeneous [9]. Lessons from previous failed trials focused on a single molecular target have led us to develop a therapeutic strategy aimed at multiple targets related to non-cell-autonomous toxicity.

Mesenchymal stem cell (MSC) therapy is a promising therapeutic strategy for addressing this issue. MSCs have been shown to exert diverse effects, such as stimulating intrinsic neurogenesis, releasing diverse neurotrophic factors, and modulating immunoinflammatory processes [10–13].

Lenzumestrocel (Korean product name; Neuronata-R® inj.) is an autologous bone marrow-derived mesenchymal stem cell and was designated as an orphan drug for concomitant therapy with riluzole in patients with ALS under the Revised Rule of Orphan Drug Designation by the Korea Ministry of Food and Drug Safety (Dec 31, 2013, MFDS Announcement No. 2013-262). In a previous phase II clinical trial, two repeated treatments with intrathecal autologous BM-MSCs (26-day interval) showed a therapeutic benefit lasting only 6 months, with good safety, in patients with ALS [14].

ALSUMMIT aims to evaluate the efficacy and long-term safety of two repeated (single-cycle) Lenzumestrocel intrathecal treatments by extending the study period. Additionally, we will assess the safety and efficacy of single-cycle + three additional booster injections. A post hoc survival analysis in our phase II trial did not show a long-term survival benefit. This may be associated with two limited injections with therapeutic effects that were not long-lasting. Considering the immunomodulatory effects of BM-MSC treatment by intrathecal delivery (less-invasive procedures), it would be essential to determine whether successive booster BM-MSC treatments after single-cycle treatment could improve long-term efficacy.

### Objectives {7}

The purpose of this study is to demonstrate the clinical superiority of Lenzumestrocel after its administration compared to a placebo. The two primary endpoints will be evaluated by comparing the therapeutic efficacy between (1) study Group 1 (single-cycle, two repeated injections of Lenzumestrocel with 26-day interval) and the control group (two administrations of the placebo) at 6 months and (2) study Group 2 (single-cycle + three additional booster injections of Lenzumestrocel) and the control group (five administrations of the placebo) at 12 months in terms of joint rank scores (JRS), also known as the combined assessment of function and survival (CAFS). The secondary endpoint will be evaluated by comparing the therapeutic efficacy between study Group 2 and the control group at 6 months in terms of JRS, change in ALSFRS-R score from baseline, and time to event at individually specified points after administration. Additionally, as the exploratory endpoint, this study will investigate pulmonary function (slow vital capacity, SVC), muscular strength (hand-held dynamometry, HHD), time-to-event, and time-to-death. Finally, serially collected biological specimens during clinical trials will be used to develop biological markers to predict the efficacy and analyse the mode of action of Lenzumestrocel.

### Trial design {8}

**Fig. 1:**
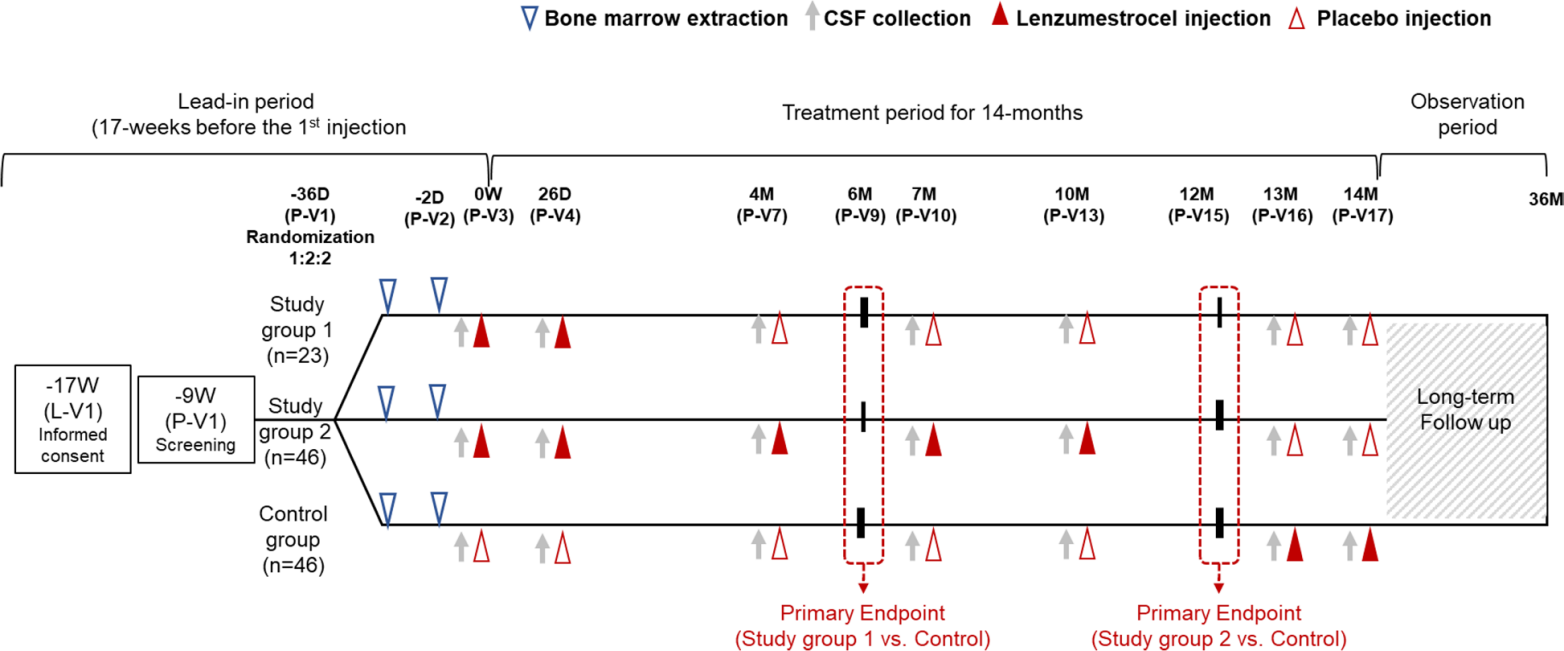
Overview of ALSUMMIT trial design. Data from study Group 1 and the control group will be analysed by JRS score at 6 months after the single-cycle treatments. Study Group 2 and the control group will be analysed by JRS score at 12 months after the combined single-cycle intrathecal therapy and three additional booster injections of MSC (Lenzumestrocel) treatment. CSF, cerebrospinal fluid; ALSFRS-R, Amyotrophic Lateral Sclerosis Functional Rating Scale-revised; JRS, joint rank score

ALSUMMIT is a multicentre, randomized, double-blind, parallel-group, sham procedure-controlled, phase III clinical trial to evaluate the efficacy and safety of Lenzumestrocel in patients with ALS. This clinical trial consists of the main study (within a 17-week lead-in period and a 14-month treatment period) and an additional observational study (Fig. 1). Among participants, patients who wish to participate in further long-term observation studies will be followed up to 3 years after the first treatment. Randomization will be conducted by the stratified block randomization method considering the stratification factors Riluzole administration (Y or N) and ALS types (bulbar or limb) to ensure that subjects are randomly assigned to study Group 1, study Group 2, and the control group in a ratio of 1:2:2.

The ALSUMMIT protocol was developed for the adoption of enrichment enrolment and add-on design and consideration of ethical issues for the placebo group.

A total of 115 subjects who meet the criteria for eligibility and the inclusion/exclusion criteria will be randomly assigned using the Interactive Web Response System (IWRS) to study Group 1, study Group 2, or the control group. Details of the eligibility and the inclusion/exclusion criteria are described in the eligibility criteria section. All subjects will be observed for 14 months after the first administration of Lenzumestrocel or placebo.

## Methods: participants, interventions, and outcomes

### Study setting {9}

The ALSUMMIT trial will be conducted in South Korea at five academic hospitals (Hanyang University Hospital, Korea University Anam Hospital, Samsung Medical Center, Seoul National University Hospital, and Pusan National University Yangsan Hospital). All data will be collected at each site.

### Eligibility criteria {10}

Participants who meet the following criteria will be included in this clinical trial:

1. Participants aged 25 to 75 years.
2. Participants showing both upper motor neuron signs (hyperactive deep tendon reflexes, positive Babinski reflex, positive ankle clonus, positive Hoffman reflex and increased muscle tone) and lower motor neuron signs (muscle wasting, muscle weakness, and fasciculation) at the time of neurological examination.
3. Participants diagnosed as familial or sporadic ALS compatible with clinically definite ALS, probable ALS, or probable ALS-lab supported, following the revised World Federation of Neurology El Escorial criteria [15].
4. Participants who show an ALSFRS-R progression rate of 1.03±0.52/month during a 17-week lead-in period prior to treatment administration.
5. Participants who have received a stable dose of riluzole for more than 28 days before the screening visit. However, it does not apply to participants whose riluzole administration is deemed impossible due to adverse events as determined by neurologists.
6. Participants have a disease duration less than 2 years from the date of initial diagnosis.
7. Participant whose ALSFRS-R score in the range of 31 to 46 at the time of screening.
8. Participants who can visit the site by themselves or with others' support.
9. Participants and/or their guardians who give consent to participate in this clinical trial.

Patients who meet the following criteria will be excluded from this clinical trial:

1. Patients who fail to satisfy the ALS diagnosis criteria, as per the revised World Federation of Neurology El Escorial Criteria.
2. Patients with primary lateral sclerosis that show only upper motor neuron signs or with progressive muscular atrophy, or low motor neuron disease that show only lower motor neuron signs.
3. Patients expected to have side effects on the administration of cell therapy (i.e. patients suspected to have malignant tumours, high-risk patients vulnerable to psychogenic shock, and severe hypertension).
4. Patients with ALSFRS-R scores of less than 31 at the time of screening.
5. Patients who received tracheostomy or use ventilators (including positive pressure ventilators; patients who use noninvasive ventilation for sleep apnoea may be allowed after review) at the time of screening.
6. Patients with gastrostomy at the time of screening.
7. Patients whose clinical efficacy evaluation will not be possible.
8. Patients who fall into or above Class II following the New York Heart Association's functional classification.
9. Patients who received other investigational products or edaravone within 3 months or 5 half-lives at the time of screening.
10. Patients who have experienced epileptic seizures.
11. Patients with severe renal disorders.
12. Patients with severe hepatic disorders.
13. Patients who have haemorrhage tendency at the time of screening.
14. Patients who are found to have active viral infections at the time of screening.
15. Patients with hypersensitivity to antibiotics.
16. Patients who have received any cell therapy product for the same disease.
17. Patients with any malignant tumour in the past 5 years before screening (except malignant tumours with low risk of metastasis or death).
18. Patients who are receiving any medicinal products that may affect bone marrow functions.
19. Patients with any neurological disease other than ALS.
20. Patients with severe mental disorders.
21. Patients under diaphragmatic pacing.
22. Patients for whom the administration of investigational products is prohibited, patients with conditions that may affect the interpretation of results or patients with conditions that may result in a high risk of complications.

### Who will take informed consent? {26a}

Informed consent will be obtained by the principal investigator or delegated sub-investigators at each site. At the beginning of the lead-in period, potential participants will be informed about this clinical trial (purpose, method, procedures, benefit, and potential hazards) and will be provided with a participant information leaflet.

In this clinical trial, several types of informed consent forms (ICFs) for the prescreening, main study, observation study, and data collection of pregnant partners and newborn babies will be prepared.

### Additional consent provisions for collection and use of participant data and biological specimens {26b}

Participants will be asked to consent to their data and biological specimens (peripheral blood, cerebrospinal fluid, bone marrow, etc.) for drug-related biomarkers study.

## Interventions

### The explanation for the choice of comparators {6b}

The control group will receive normal saline (sterilized isotonic sodium chloride solution). Intrathecal administration of normal saline is the most common comparator used in clinical trials for stem cell therapy in neurological disease (NCT03355365, NCT01254539, NCT04749667, NCT04528550, NCT03521323). It has been reported that there are no serious adverse effects, no cells, and it has similar compositions to human body fluid [16].

### Intervention description {11a}

The 115 subjects will be randomized (1:2:2) into three groups: (1) study Group 1 (*n*=23, two injections of Lenzumestrocel at 0 and 26 days and followed by three injections of placebo at 4, 7, and 10 months), (2) study Group 2 (*n*=46, five injections of Lenzumestrocel at 0 and 26 days, 4, 7, and 10 months), and (3) control group (*n*=46, five injections of placebo at 0 and 26 days, 4, 7, and 10 months).

After the initial two injections of Lenzumestrocel or placebo with 26-day intervals, additional three administrations with 3-month intervals will be provided. To determine the potential benefit of three additional Lenzumestrocel treatments and an optimal interval, we will analyse serial ALSFRS-R scores and CSF biomarkers in postmarketing surveillance data. When comparing different treatment intervals (3 to 4, 6 to 8, 12 months), the clinical benefits on ALSFRS-R will be maximized after additional regular treatment with 3- to 4-month intervals, which is consistent with the CSF inflammatory cytokine level findings. Using these data, this phase III clinical trial to confirm the long-term efficacy and safety of Lenzumestrocel will be developed and finally approved by the U. S FDA. To avoid the ethical issue the control group faces, who cannot receive any active treatment during the clinical trial despite the two bone marrow aspirations, all participants will receive two additional intrathecal treatments with a 1-month interval. The purpose of this 6th and 7th intrathecal treatment (at 13 and 14 months after the first administration) is the ethical consideration of the control group. Clinical data will not be opened, and clinical trials will be continued in a blinded state for 2 months until the completion of two Lenzumestrocel treatments for the control group.

Lenzumestrocel is an autologous bone marrow-derived mesenchymal stem cell (BM-MSC) that was isolated, expanded, and analysed under good manufacturing practice (GMP) conditions at Corestem Inc. (Seoul, Korea), based on the International Society of Cellular Therapy guidelines [17]. Details of the manufacturing procedures of Lenzumestrocel are described in our previous phase II paper [14].

Lenzumestrocel will be supplied as a 4 mL MSC cell suspension (1×10^7^ cells per mL of CSF) and prefilled in a syringe. For the allowance of sufficient time for ex vivo MSC expansion, bone marrow extraction will be performed 36 days before the first Lenzumestrocel injection. One day before Lenzumestrocel injection, participants’ own CSF will be collected for use as a suspension. Lenzumestrocel will be delivered to the hospital and will be administered to the participant within 48 h from the completion of the suspension.

Using a standard lumbar puncture at the level of L2–L4, Lenzumestrocel will be slowly injected (total number of MSCs adjusted by body weight, 1×10^6^ cells per kg) intrathecally over approximately 2 minutes. Subsequently, participants will remain in the Trendelenburg position with the application of a mechanical vibrator on their hip bone for 2 h.

All participants will receive continuous riluzole treatment (100 mg/day) except those with side effects. All subjects will be observed for 14 months after the first administration of Lenzumestrocel or placebo.

### Criteria for discontinuing or modifying allocated interventions {11b}

Participants who have withdrawn from the study cannot participate in this study again, but there is no disadvantage due to the withdrawal of the clinical trial for their medical care. Allocated interventions cannot be modified.

Participants may discontinue from the study at any time and for any of the following reasons:

1. Withdrawal of the informed consent
2. Death of the participant
3. The decision of the principal investigator or sub-investigator because of safety issues

If the participant does not wish to administer the study drug but to continue participating in the study, it is regarded as the end of treatment, and follow-up is continued.

### Strategies to improve adherence to interventions {11c}

To prevent visit deviation and ensure that the investigational product can be administered within the approved period, the research staff will contact the subjects to inform them of the visit date in advance (2 weeks, 1 week, 1 day). Upon receiving consent for participation, investigators should try to obtain at least two or more contact points to stay in touch with the subjects. In addition, as ALS patients are at high risk of accidents (e.g. fall, aspiration) due to carelessness, so investigators shall thoroughly conduct safety education to subjects and guardians to prevent accidents.

### Relevant concomitant care permitted or prohibited during the trial {11d}

All participants will receive a standard dose of riluzole except for side effects, and symptomatic treatments are also allowed in all groups.

The following medication will be permitted concomitantly administered during this trial.

1. Any medication taken by a subject before their participation in this clinical trial and is considered not to affect the interpretation of these study results can be allowed as determined by the investigator.
2. Any medicinal products transiently used to treat other diseases or adverse events can be allowed after consulting with the investigator.

The following medications that may interfere with the outcome of this trial will be prohibited during this trial.

1. Yoo solution (ursodeoxycholic acid) for treatment of ALS
2. Radicava® (edaravone)
3. Any stem cell products except for Lenzumestrocel
4. Other Investigational products indicated for ALS treatment
5. Herbal medicines (such medicines are not completely characterized and may cause toxic effects or unexpected drug interactions that can adversely affect the efficacy and safety evaluation)
6. Nuedexta® (dextromethorphan and quinidine)
7. Ketas® (Ibudilast)

If concomitant medication is necessary for the medical treatment of a subject, as determined by the investigator or a medical doctor, the medicine may be used during the clinical study period (from lead-in period L-V1).

### Provisions for post-trial care {30}

To provide direct benefit from the investigational drug, participants who are allocated to the control group will receive Lenzumestrocel after the primary endpoint (at 13 and 14 months after the first administration).

In compliance with the compensation criteria for subjects, the sponsor should keep the subjects compensated for any injury that occurred either by an adverse event, investigational products, or treatment that attributes directly to the investigational products.

### Outcomes {12}

The two primary endpoints are a joint-rank test of function (ALS Functional Rating Scale-Revised) and overall survival (Table 1). The joint-rank test is a robust endpoint for ALS studies because of the ability to interpret death and functional change [18].

**Table 1:** Description of primary, secondary, exploratory endpoints and analysis method

Endpoints	Description	Analysis method
Primary	(1) Difference in joint rank scores between study Group 2 and control group at 12 months (2) Difference in joint rank scores between study Group 1 and control group at 6 months	(1, 2) Analysis of covariance (ANCOVA) with adjustment of baseline ALSFRS-R score, baseline ALSFRS-R progression rate, and stratification factors (1, 2) Generalized Gehan-Wilcoxon rank test for supportive analysis
Secondary	(1) Difference in joint rank scores between study Group 2 and control group at 6 months (2) Change from baseline in ALSFRS-R score at 12 months (study Group 2 and control group) (3) Change from baseline in ALSFRS-R score at 6 months (study Group 1 and control group)	(1, 2, 3) ANCOVA with adjustment of baseline ALSFRS-R score, baseline ALSFRS-R progression rate, and stratification factors
	(4) Time-to-event at 12 months (study Group 2 and control group) (5) Time-to-event at 6 months (study Group 1 and control group)	(4, 5) Stratified cox proportional hazards model with adjustment of baseline ALSFRS-R progression rate and stratification factors
Exploratory	(1) Comparison of the change from baseline in SVC score at 6 months (study Group 1 and control group) and 12 months (study Group 2 and control group)	(1, 3, 8, 10) ANCOVA with adjustment of the baseline value, baseline ALSFRS-R progression rate, and stratification factors
	(2) Change from baseline in SVC score at 36 months (3) Comparison of the change from baseline in muscular strength at 6 months (study Group 1 and control group) and 12 months (study Group 2 and control group) (4) Change from baseline in muscular strength at 36 months (5) Time-to-event at 36 months (6) Comparison of the time-to-death at 6 months (study Group 1 and control group) and 12 months (study Group 2 and control group) (7) Time-to-death at 36 months (8) Comparison of the change from baseline in EQ-5D-5 L index value at 6 months (study Group 1 and control group) and 12 months (study Group 2 and control group) (9) Change from baseline in EQ-5D-5 L index value at 36 months (10) Comparison of the change from baseline in ALS Assessment Questionnaire (ALSAQ)-40 scores at 6 months (study Group 1 and control group) and 12 months (study Group 2 and control group) (11) Change from baseline in ALSAQ-40 scores at 36 months (12) Comparative analysis of cytokines in peripheral blood at baseline and individual points (13) Comparative analysis of cytokines in cerebrospinal at baseline and individual points (14) Analysis of regulatory T cell functions at baseline and individual points (15) Comparative analysis of transcriptome at baseline and individual points	(2, 4, 5, 7, 9, 11) Summarized by treatment groups
		(6) Stratified cox proportional hazards model with adjustment of baseline ALSFRS-R progression rate and stratification factors
		(12, 13, 14) Repeated measures generalized estimating equations with an AR(1) correlation structure
		(15) Standard transcriptome analysis pipeline

The first primary endpoint will be analysed at 6 months, comparing study Group 1 to the control group. The second primary outcome will be analysed at 12 months, comparing study Group 2 to the control group. The ALSFRS-R score (48 [normal] to 0 [maximally impaired]) will be assessed throughout a 3-month lead-in period and 12-month follow-up period, as shown in Fig. 1.

Secondary endpoints are a joint rank test at 6 months comparing study Group 2 to the control group, change from baseline in ALSFRS-R score at 6 months (comparing study Group 1 to control group), and again at 12 months (comparing study Group 2 to control group) (Table 1). Additional secondary endpoints are the time-to-event at 6 and 12 months.

The time until physical death or tracheostomy is recognized as an event where the disease progression functionally stops or the chronic use of a ventilator (chronic assisted ventilation; use of noninvasive ventilation for more than 20 h a day for 30 consecutive days or more), whichever comes earlier, subjects are observed for 6 months and 12 months after randomization.

Exploratory endpoints are listed in Table 1.

### Participant timeline {13}

Participant timeline is shown in Additional file 1.

### Sample size {14}

Sample size was calculated based on the previous phase I/II clinical trial (Table 2). Two hypotheses employed in this clinical study are as follows:

**Table 2:** CAFS results (mean and standard deviation) at 6 and 12 months in phase I/II clinical trials

Point	Group	*N*	CAFS, mean	CAFS, SD
6 months	Study group	39	40.79	17.52
	Control group	27	22.96	16.56
12 months	Study group	39	39.14	18.22
	Control group	27	25.35	17.82

Hypothesis 1.*P*(uc_6_ < ut_6_) = 0.5 vs. *P*(uc_6_ < ut_6_) ≠ 0.5

Hypothesis 2.*P*(uc_12_ < ut_12_) = 0.5 vs. *P*(uc_12_ < ut_12_) ≠ 0.5

where

μc_6_: control group’s mean CAFS at 6 months

μt_6_: study Group 1’s mean CAFS at 6 months

μc_12_: control group’s mean CAFS at 1s2 months

μt_12_: study Group 2’s mean CAFS at 12 months

Accordingly, 1:1 of study Group 1 and control group, a two-sided significance level of 0.05 and statistical power of 80% were assumed for Hypothesis 1, and 1:1 of study Group 2 and control group, a two-sided significance level of 0.05 and statistical power of 80% were assumed for Hypothesis 2. For each hypothesis, the minimum number of subjects required to demonstrate that the study group showed superior results to the control group was calculated according to the following equation:

$$N=\frac{{\left({Z}_{\alpha }+{Z}_{\beta}\right)}^2}{12c\left(1-c\right){\left(p"-\frac{1}{2}\right)}^2}$$

two-sided significance level (α) = 0.05

statistical power (1-β) = 0.80

c = ratio of subjects in the study group relative to all subjects = 0.5

$$p"=\Pr \left({\mathrm{uc}}_{\mathrm{m}}<{\mathrm{ut}}_{\mathrm{m}}\right) if\ m= 12, 0.705; m= 6, 0.769$$

Based on the CAFS data obtained in phase I/II clinical trials, the number of subjects was calculated for two hypotheses to demonstrate the study group's superiority to the control group at 6 and 12 months. Table 3 shows the estimated sample size of this clinical study. For Hypothesis 1, the calculated number of subjects in study Group 1 is 19, and the calculated number of subjects in the control group is 19. For Hypothesis 2, the calculated number of subjects in study Group 2 is 32, and the estimated number of participants in the control group is 32. Since a single control group was used for both study Group 1 and study Group 2 in this clinical study, a larger number of subjects (32) in the control group for two hypotheses was selected. In conclusion, the minimum number of subjects required for this clinical study was 19 for study Group 1, 32 for study Group 2, and 32 for the control group. Considering the 15% (around) dropout rate, the number of subjects was increased to 23 for study Group 1, 38 for study Group 2, and 38 for the control group. However, to satisfy the stratified block randomization, a randomization ratio was generated at 1:2:2 for each group. Therefore, to satisfy the ratio of 1:2:2, 23 subjects in study Group 1, 46 subjects in study Group 2, and 46 subjects in the control group will be enrolled (115 subjects in total).

**Table 3:** Estimated sample size considering dropout rate

	Study Group 1	Study Group 2	Control group	Total No. of subjects
Cases for efficacy evaluation	19	32	32	83
Cases including dropouts (approximately 15%)	23	46	46	115

### Recruitment {15}

We will promote the trial progress to Korean ALS associations and provide trial information to physicians who care for ALS patients to guide patients interested in the trial to transfer to the investigational site. The investigators at each site will faithfully perform the participant screening. Externally, we continue to promote the clinical trial by recruiting participants who meet the qualifications for the clinical study. Participants are given a detailed explanation of the clinical trial and enough time to consider participating in the clinical trial. Administrators continuously communicate with the trial participants and faithfully address any questions or opinions.

## Assignment of interventions: allocation

### Sequence generation {16a}

Randomization will be conducted by the stratified block randomization method (in consideration of stratification factors; Riluzole administration (Y or N) and ALS type (bulbar or limb)) to ensure that subjects are randomly assigned to study Group 1, study Group 2, and the control group at a ratio of 1:2:2. Randomization numbers are generated by an independent statistician of this clinical study using SAS (version 9.4 or higher, SAS Institute, Cary, NC, USA).

Subjects enrolled in the clinical study and satisfying the inclusion/exclusion criteria will be sequentially assigned to individual groups through the web-based automatic response service (Interactive Web Response System, IWRS) according to their randomization numbers. Such randomization numbers are used as subject identification codes during the clinical study period.

### Concealment mechanism {16b}

The randomization number generated through IWRS is e-mailed to the delegated staff, including investigators. The randomization number is sequenced so that the allocation information cannot be known, and therefore, no one knows the participants’ intervention arm. The unblinded staff who will be limitedly delegated to know the allocation information must access and check the RTSM (randomization and trial supply management) system.

The generation of the allocation sequence will be conducted by an independent statistician who is not involved in other clinical trial procedures. The investigators will enrol the participants.

## Assignment of interventions: blinding

### Who will be blinded {17a}

During the study period, the investigators, the study participants, all study site personnel (except for the site-specific unblinded staff in charge of storage, management, and administration of the investigational products), and data management personnel who will be involved in data cleaning and analysis of the data will be blinded to the treatment group assignment. Treatment unblinding for the study will occur after all clinical data have been received, data inconsistencies have been resolved, and the database is locked, except for safety reasons on a case-by-case basis (e.g. emergency unblinding).

### Procedure for unblinding if needed {17b}

Premature breaking of the blind will be allowed only if the subject’s well-being requires knowledge of the treatment allocation. Every attempt will be made to maintain blindness throughout the study.

In the event of an urgent safety issue where the randomized treatment of a subject is necessary to manage and treat the affected study subject (e.g. unblinding subjects due to SAEs that meet “expedited criteria” and require reporting to regulatory authorities), emergency unblinding is unavoidable. The principal investigator may consult with the sponsor. However, the principal investigator may perform code-breaking at their discretion if such consultation is not available or in an urgent situation.

The reason for unblinding must be recorded. However, the investigator must not record the subject treatment assignment in the study documentation and must not reveal the subject treatment assignment.

## Data collection and management

### Plans for assessment and collection of outcomes {18a}

Efficacy data (i.e. ALSFRS-R, SVC, FVC, and HHD) will be collected by blinded research staff who are certified after rater training. Data will be collected and managed via the Medidata Rave electronic data capture (EDC) system. Sites oversee data input on the EDC system.

### Plans to promote participant retention and complete follow-up {18b}

There are several alerting systems to keep the planned assessment or procedure schedule. The investigational product order system developed for the study will send a text to the research staff 1 week and 1 day before the planned date to contact the participants to remind them of their schedule. In addition, participants will repeatedly receive the reminder texts through the alerting system of each site at a set time before the planned schedule. Remote evaluation (ALSFRS-R, time-to-event/death, ALSAQ-40, and EQ-5D-5 L) can be conducted for subjects who do not withdraw their consent even though the clinical study is discontinued.

### Data management {19}

Investigators will conduct assessments and record them in the source data. Designated research staff will transcribe the data from source data into EDCs. We have delegated a series of processes related to data management to the CRO (clinical research organization) and reviewed these processes as a sponsor. Review and cleaning of data entered in EDC will be performed by CDA (Clinical Data Analyst). The potential data errors identified by triggered edit checks will prompt CDA to perform data-cleaning activities such as generating queries to a site.

Data review will be performed by study CDAs using the following documents:

1. The Data Validation Specification (DVS): The document defines the programmed checks applied to the clinical data within the EDC.
2. CRF Completion Instruction (CCI): This document provides the study investigators and the site staff with general guidelines for accessing and completing eCRFs. CCI describes the specific requirements used for completing paper CRFs and entering data into electronic CRFs.
3. Offline Listing Mock shell: This document provides the manual view of study data via SAS listings and reports.
4. Data Acquisition and Entry Specification: This document provides detailed information on handling other paper components received from the site for data entry into EDC.

### Confidentiality {27}

The sponsor shall identify the subjects in encrypted ID numbers for confidentiality; in ways, the name of subjects is not exposed in any data provided to it. Subjects’ medical information obtained during the clinical trial is confidential. Unless otherwise legally permitted or requested, such information may only be provided to third persons in ways permitted under (either) an informed consent form (or subjects' consent for use and publication of personal health information). However, such medical information may from time to time be provided to medical practitioners treating the subject or to any other medical practitioners deemed appropriate.

If requested, the clinical trial results may be provided to the persons in charge of local or international health authorities, monitors, staff, and assistants from the sponsor and the site IRBs for investigation purposes.

Plans for collection, laboratory evaluation, and storage of biological specimens for genetic or molecular analysis in this trial/future use {33}

Laboratory tests will be conducted at the clinical laboratory at each site according to the clinical study schedule for all subjects to evaluate overall health conditions. In addition, pregnancy tests and laboratory tests will be conducted before the administration of investigational products to verify the entire satisfaction of the inclusion/exclusion criteria. Laboratory tests for screening conducted at P-V0 (haematological tests, blood chemistry tests, urinalysis, blood coagulation tests) may be re-examined once based on the investigator's decision. Subject eligibility will be evaluated following the final examination results. Subjects who satisfy the inclusion and exclusion criteria at L-V1, L-V2, and P-V0 will be randomly assigned. This will be followed by the collection of bone marrow, according to the predetermined methods at P-V1.

In addition, to prepare the investigational products, CSF will be collected 1~2 days before administering investigational products. The bone marrow and CSF of subjects who have signed informed consent forms for human-derived materials can be stored for years, or as long as the subject wants, to improve quality control and research.

A study of biomarkers deemed necessary by the investigator may be conducted in addition to tests on clinical study schedules. Genetic testing samples for the ALS-related genes (*SOD1*, *TDP-43*, and *C9ORF72*) are submitted to the external central laboratory. Tests on blood and CSF samples for exploratory biological markers will be conducted only for those who gave informed consent in advance for additional research. Moreover, if the subject signed the informed consent form for human-derived materials, the remaining specimen can be stored for further study.

## Statistical methods

### Statistical methods for primary and secondary outcomes {20a}

All inferential statistical analyses will be based on a two-sided test with a type I error rate of 0.05.

The two primary efficacy endpoints for this study are as follows:

1. Joint rank test using CAFS between study Group 2 and the control group at 12 months
2. Joint rank test using CAFS between study Group 1 and the control group at 6 months

To protect the type I error rate for testing the two hypotheses, a hierarchical test procedure will be used. The hypotheses will be tested in the following order:

1.

H_0_: No difference between study Group 2 and the control group at 12 months.

H_1_: There is a difference between study Group 2 and the control group at 12 months.

2.

H_0_: No difference between study Group 1 and the control group at 6 months

H_1_: There is a difference between study Group 1 and the control group at 6 months.

If the first null hypothesis is rejected at an alpha level of 0.05, then study success is claimed. Next, the second null hypothesis is tested at an alpha level of 0.05. If the first null hypothesis is not rejected at an alpha level of 0.05, then the study fails.

At individual points, the differences between the study and control groups will be evaluated by analysis of covariance (ANCOVA) with covariate adjustment of baseline ALSFRS-R score, ALSFRS-R score progression rate, and stratification factors. When a 95% two-sided confidence interval for differences in mean joint rank scores between the study and control groups at each point does not comprise 0, it is considered that the study group's results are superior to those of the control group.

Additionally, for the primary efficacy endpoints, a generalized Gehan-Wilcoxon rank test will be used to compare CAFS between study groups and control groups as a supportive analysis.

The secondary efficacy endpoints for this study are as follows:

1. Joint rank test using CAFS between study Group 2 and the control group at 6 months
2. Change in ALSFRS-R score from baseline between study Group 2 and the control group at 12 months
3. Change in ALSFRS-R score from baseline between study Group 1 and the control group at 6 months
4. Comparison of the time to an event at 6 months between study Group 1 and the control group
5. Comparison of the time-to-event at 12 months between study Group 2 and the control group

The joint rank test is calculated in the same way as the primary outcome. The effect of the treatment in terms of the change from baseline in ALSFRS-R scores will be analysed using ANCOVA with covariate adjustment of baseline ALSFRS-R score, baseline ALSFRS-R score progression rate, and stratification factors. The effect of treatment in terms of time-to-event will be analysed using the stratified Cox proportional hazard model with covariate adjustment of baseline ALSFRS-R progression rate and stratification factors.

### Interim analyses {21b}

Not applicable. This clinical study does not plan to conduct an interim analysis.

### Methods for additional analyses (e.g. subgroup analyses) {20b}

The uniformity of the treatment effect for the primary efficacy outcome will be examined for the following subgroups using all analysis sets.

1. Age group (<54, ≧54 years)
2. Sex (Male, female)
3. Race (Korean, non-Korean) (Note that this variable will not be analysed if the numbers of non-Korean are low)
4. ALS type (Bulbar, limb)

The homogeneity of the treatment effect across subgroups will be investigated using analytical methods. Statistical tests for the presence of a treatment-by-subgroup interaction will be performed by including the interaction term in the primary analysis model. If the treatment-by-subgroup interaction is found to be statistically significant at the 10% level, this will be taken as evidence of heterogeneity of the treatment effect across subgroups.

### Methods in analysis to handle protocol non-adherence and any statistical methods to handle missing data {20c}

Data will be analysed using the intention-to-treat (ITT) set. This set will be applied to all randomized subjects. Subjects will be randomized and analysed for efficacy evaluation.

For the primary and secondary efficacy endpoints, multiple imputation (MI) methods will be used to handle the missing values, if applicable. The multiple imputation method replaces each missing value with a set of plausible values that represent uncertainty and imputation with the right value. The method for imputing variables is available in SAS PROC MI for both monotone and arbitrary missing data patterns and will be implemented with the PREDICT MEAN MATCHING option. In addition, sensitivity analyses will be conducted for the primary efficacy endpoint to examine the impact of missing data using the SAS PROC MI for both monotone and arbitrary missing data patterns using the REGRESSION option.

### Plans to give access to the full protocol, participant-level data and statistical code {31c}

Not applicable. There are no plans for granting public access to the full protocol, participant-level dataset, or statistical code.

## Oversight and monitoring

### Composition of the coordinating centre and trial steering committee {5d}

The Clinical Research Organization (CRO) is designated for clinical operation (including periodic site monitoring), data management, safety reporting management, medical oversight, data analysis and medical writing; therefore, a weekly/biweekly meeting will be held during the clinical trial process with the key person of CRO, and a monthly/quarterly report on each item will be received. In this process, only blinded staff will participate, and unblinded items will not be included in all meetings and reports. If necessary, unblinded staff will hold meetings, and unblinded reports will be forwarded to only the unblinded project manager.

The trial steering committee has executive authority regarding the study. The trial steering committee is composed of the Chief Executive Officer (CEO), Chairman, and Chief Financial and Marketing Officer (CFO) of Corestem, who are not associated with the execution of the study. Steering committee meetings will be held every week, and the project leader will report the progress of the clinical trial to the committee. The trial steering committee will give the final decision for the necessary funding, finances, and recruitment for the study. It is the committee's role to assign IDMC members and to make a final decision upon receiving the recommendations from IDMC.

### Composition of the data monitoring committee, its role and reporting structure {21a}

The IDMC consists of 3 independent experts in the fields of neurology and biostatistics. IDMC members and their contact information are provided, and they shall have no substantive conflict of interest with the study. IDMC is primarily responsible for reviewing the safety data and evaluating the study progress. Additionally, IDMC provides recommendations to the sponsor concerning continuation, termination, or other modifications of the study based on the observed adverse effects of the treatment under investigation. The operation of IDMC shall comply with the predetermined IDMC Charter that sets forth the members, roles, responsibilities, and communication affairs. IDMC shall convene at least three rounds of meetings (after 28 randomized subjects complete the main study, after an additional 56 randomized subjects complete the main study, and after 14 randomized subjects complete the main study and the observational study) to review the benefit-risk data.

During the trial, IDMC will be responsible for periodically reviewing the study results, evaluating the treatment groups for excess adverse events, determining whether the basic study assumptions remain valid, assessing whether the overall integrity, scientific merit, and conduct of the study remain acceptable, and making recommendations to the sponsor.

### Adverse event reporting and harms {22}

Subjects are instructed to report any adverse events voluntarily, and investigators are responsible for identifying adverse events at regular or additional visits through interviews or medical examinations. Information on adverse events, such as occurrence date, disappearance date, severity, and consequence of the adverse event, actions were taken regarding investigational products, causal relationship with investigational products/concomitant medication, suspected cause other than investigational products and concomitant medication, treatment of the adverse event, and others will be collected and recorded. To identify acute adverse events after administration of investigational products, subjects will be monitored for blood pressure, pulse, body temperature, and respiration at approximately 6-hour intervals for at least 24 h after administration of investigational products. A serious adverse event must be reported to the sponsor within 24 h from the time of the event and acknowledged by the investigator. In addition, especially significant adverse events as defined below will be collected and reported to the sponsor immediately (within 24 h).

Suspected transmission from an infection source by the investigational product as defined below is also considered an especially significant adverse event.

**Fig. 2:**
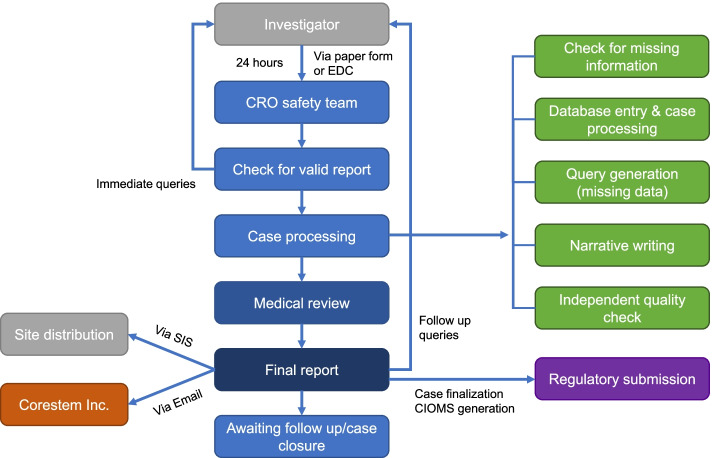
Safety management workflow. SIS, safety information system

Organisms, viruses, or infectious particles are considered infection sources regardless of pathogenicity. Transmission from an infection source is suspected if a subject exposed to drugs has clinical symptoms or obtains laboratory examination results that imply infection. The term especially significant adverse event is only used when investigational product contamination is suspected. Detailed reporting of serious adverse events is shown in Fig. 2.

### Frequency and plans for auditing trial conduct {23}

At least one audit is planned for all five sites to review the clinical study data, source document, and eCRF, which will be conducted by an independent agency delegated by the sponsor.

### Plans for communicating important protocol amendments to relevant parties (e.g. trial participants, ethics committees) {25}

When obtaining approval for a clinical trial or a change of approved clinical trial is needed, the related protocols and comparison table for change will be approved by the IRB and, if required, to the regulatory authorities. Unless a revision of the clinical trial protocol is desperately needed to eliminate the immediate risks posed on the subjects or is related to logistics or operations (such as changes in medical monitors and contact information), then a revision of the clinical trial protocol must be approved by both the IRB and the regulatory authorities. An informed consent form must be revised every time the clinical trial process is updated, or any new information that may affect the subject's decision to participate in this clinical trial becomes available. The IRB-approved final informed consent form shall be provided to the sponsor for submission to the regulatory authorities.

### Dissemination plans {31a}

Regardless of the results of this clinical trial, the sponsor will disclose the information of the clinical trial to medical practitioners and the general public through academic conferences or journals. The sponsor will comply with all requirements for the publication of clinical trial results. By signing this clinical trial protocol, the investigator agrees to use the results of this trial for the purposes of registration, presentation, and provision of information for medical/pharmaceutical experts. The sponsor shall have a right to review the clinical trial results to be declared to any journal in advance. Upon completion of clinical trial data analyses at all sites, the sponsor will keep the investigators notified of such results in the form of a report.

The sponsor shall have exclusive ownership of all data and results derived from and a right to publish the results of this clinical trial. In no case may the investigator publish, declare, present, or make public any part of the results of this clinical trial, unless otherwise agreed in writing by the sponsor. As such, the sponsor shall also ensure that the investigators do not publish, declare, present, or make public the results. To use only accurate and verified data, the investigator must provide all published drafts or presentation manuscripts prepared before publication or presentation to the sponsor for discussion, and the presentation must be withheld until written approval is received.

Regarding the clinical study results, the investigator agrees not to publish the results of their institution or some institutions before announcing the results collected from all clinical trial institutions. However, there are exceptions in cases where all institutions' clinical investigators and sponsor officially recognize the results.

## Discussion

Currently, only two drugs have been approved to be effective in ALS, riluzole, and edaravone, which show only modest effects on disease progression. There have been several recent scientific advances in cell-based therapies that might translate into novel therapeutic strategies for ALS. Prior to our phase I and II trials, Lenzumestrocel and BM-MSCs were shown to be feasible and clinically beneficial for functional decline in ALS [14, 19]. However, there is currently no confirmatory evidence for the effect of stem cell-based therapy on the course of disease in patients with ALS [20]. ALSUMMIT is one of 2 phase III trials registered on ClinicalTrials.gov (as of Sept 27, 2021) to test this hypothesis.

ALSUMMIT has several advantages compared to previous clinical trials of stem cell therapies for ALS. First, this study was a double-blinded trial and included a sham procedure in the control group. There are only two sham procedure-controlled clinical trials registered on ClinicalTrials.gov (as of Sept 27, 2021). In previous phase I and II clinical trials, two repeated intrathecal injections of BM-MSCs were safe and well-tolerated. When the trial includes a sham procedure-controlled arm, the risks to participants can be increased. However, the inclusion of a sham procedure arm is essential to minimize bias and confounders in confirmatory late-stage clinical trials [21]. Second, the follow-up period was at least 12 months postinjection. The FDA has recommended a clinical trial duration of 6 to 12 months to assess clinical efficacy [22]. Thus, ALSUMMIT can establish the long-term effectiveness of BM-MSC treatment. Third, enrichment strategies were applied to ALSUMMIT design. It is crucial to reduce clinical or biological heterogeneity when enrolling subjects and post hoc analysis on biological markers to identify the subgroup of patients who appear to respond better to the specific treatment. Participants who have an intermediate rate of disease progression will be included in this trial to reduce clinical heterogeneity. Several clinical trials applied prognostic or predictive enrichment strategies, including a progression rate calculated by ALSFRS-R, disease onset, serum urate, FVC, and C-reactive protein (CRP) [23]. Studies of biomarkers of CSF and serum inflammatory cytokines, mRNA, and variants in ALS-related genes in this trial can help identify a subgroup with good effectiveness. Fourth, ALSUMMIT is an add-on design. All participants in this trial will receive the standard of care, including riluzole. Considering the rapid progression and fatality of ALS, it would be unethical to give control groups no investigational products that may have clinical benefit. Fifth, to reduce incorporation and loss of opportunity for potential treatment in sham procedure-controlled groups, the control group will receive Lenzumestrocel after the primary endpoint. Sixth, MSC cryopreservation in this trial allows two or more repeated administrations, even from a single bone marrow extraction. Frozen MSCs have a 2-year shelf life and can be used in Lenzumestrocel production without additional bone marrow extraction for repeated administrations.

Although the sample size was calculated considering the usual dropout rate in clinical trials, the ALSUMMIT trial protocol was designed to require a long-term follow-up time of up to 12 months. In the previous phase II clinical trial, the primary outcome was determined 6 months after the first treatment. Therefore, a higher dropout rate may be developed in the ALSUMMIT trial. Multiple imputations will estimate the missing value, but if the dropout rate is higher than the initial expectation and appropriate data analysis is difficult with high dropout rate, additional recruitment of new participants or addition of a new treatment arm could be considered after the discussion with FDA and MFDS.

We hope that data from the ALSUMMIT trial would contribute to developing the BM-MSC therapeutic strategy for ALS.

## Trial status

The trial was approved by the U.S FDA and South Korea Ministry of Food and Drug Safety (MFDS) on Jul 24, 2020, and Aug 26, 2020, respectively. The current protocol version is 8.0. Recruitment is currently ongoing. Participant enrolment started on Mar 24, 2021, and the estimated end date of recruitment is Nov 30, 2022.

## Supplementary Information

**Additional file 1.**

## Abbreviations

ALS: Amyotrophic lateral sclerosis
ALSAQ: Amyotrophic Lateral Sclerosis Assessment Questionnaire
ALSFRS-R: Amyotrophic Lateral Sclerosis Functional Rating Score-Revised
ANCOVA: Analysis of covariance
CAFS: Combined assessment of function and survival
eCRF: Electronic case report form
EDC: Electronic data capture
HHD: Hand-held dynamometry
IWRS: Interactive Web Response System
JRS: Joint rank score
SVC: Slow vital capacity
