# Supplementary material for: Efficacy and safety of Lenzumestrocel (Neuronata-R® inj.) in patients with amyotrophic lateral sclerosis (ALSUMMIT study): study protocol for a multicentre, randomized, double-blind, parallel-group, sham procedure-controlled, phase III trial
Source: Trials. 2022 May 18;23:415. doi: 10.1186/s13063-022-06327-4 (PMC9115933; doi:10.1186/s13063-022-06327-4)
Supplement: Supplementary file 1 — Additional file 1. [file 13063_2022_6327_MOESM1_ESM.doc]

Supplementary Table 1. Main study schedule

| **Visit** | **Lead in Period** | | | | | | | **Treatment Period** | | | | | | | | | | | | | | | | | | | | | | | | | | | | | | | |
| --- | --- | --- | --- | --- | --- | --- | --- | --- | --- | --- | --- | --- | --- | --- | --- | --- | --- | --- | --- | --- | --- | --- | --- | --- | --- | --- | --- | --- | --- | --- | --- | --- | --- | --- | --- | --- | --- | --- | --- |
| **Day, Week, Month** | **-17 W** | **-13 W** | | **-9 W** | | **-36D** | | **-3D**  **~**  **-2D** | | **0D** | | **26D** | | **8W** | | **12W** | | **16W** | | **20W** | | **24W** | | **28W** | | **32W** | | **36W** | | **40W** | | **44W** | | **48W** | | **52W** | | **56W** | |
| Informed consent | X |  | | X | |  | |  | |  | |  | |  | |  | |  | |  | |  | |  | |  | |  | |  | |  | |  | |  | | X | |
| Eligibility screening | X | X | | X | |  | |  | | X | |  | |  | |  | |  | |  | |  | |  | |  | |  | |  | |  | |  | |  | | X | |
| Randomization |  |  | |  | | X | |  | |  | |  | |  | |  | |  | |  | |  | |  | |  | |  | |  | |  | |  | |  | |  | |
| **Assessment (Efficacy & QoL, safety)** | | | | | | | | | | | | | | | | | | | | | | | | | | | | | | | | | | | | | | | |
| ALSFRS-R^2)^ | X | | X | | X | | X | |  | | X | | X | | X | | X | | X | | X | | X | | X | | X | | X | | X | | X | | X | | X | | X |
| SVC, HHD |  | |  | | X | |  | |  | | X | | X | |  | |  | | X | |  | | X | |  | | X | |  | | X | |  | | X | |  | | X |
| Time to event |  | |  | | X | | X | | X | | X | | X | | X | | X | | X | | X | | X | | X | | X | | X | | X | | X | | X | | X | | X |
| ALSAQ-40,  EQ-5D-5L |  | |  | | X | |  | |  | | X | |  | | X | |  | | X | |  | | X | |  | | X | |  | | X | |  | | X | |  | | X |
| VS, BW, Ht, PE, ECG, CXR |  | |  | | X | | X | | X | | X | | X | | X | | X | | X | | X | | X | | X | | X | | X | | X | | X | | X | | X | | X |
| Pregnancy test |  | |  | | X | |  | | X | |  | |  | |  | |  | |  | |  | | X | |  | |  | |  | |  | |  | | X | |  | | X |
| Laboratory test |  | |  | | X | |  | |  | | X | |  | |  | |  | |  | |  | | X | |  | |  | |  | |  | |  | | X | |  | | X |
| AEs, COM |  | |  | | X | | X | | X | | X | | X | | X | | X | | X | | X | | X | | X | | X | | X | | X | | X | | X | | X | | X |
| **Procedures & Intervention** | | | | | | | | | | | | | | | | | | | | | | | | | | | | | | | | | | | | | | | |
| CSF collection & test |  | |  | |  | |  | |  | | X | | X | |  | |  | | X | |  | |  | | X | |  | |  | | X | |  | |  | | X | | X |
| BME |  | |  | | X | | X | |  | |  | |  | |  | |  | |  | |  | |  | |  | |  | |  | |  | |  | |  | |  | |  |
| IP injection |  | |  | |  | |  | |  | | X | | X | |  | |  | | X | |  | |  | | X | |  | |  | | X | |  | |  | | X | | X |
| **Exploratory study** | | | | | | | | | | | | | | | | | | | | | | | | | | | | | | | | | | | | | | | |
| MOA (CSF) |  | |  | |  | |  | |  | | X | | X | |  | |  | | X | |  | |  | | X | |  | |  | | X | |  | |  | | X | | X |
| MOA (PB) |  | |  | |  | |  | |  | | X | | X | | X | |  | | X | | X | |  | | X | | X | |  | | X | | X | |  | |  | |  |

D=day, W=week, QoL=Quality of life, ALSFRS-R=Amyotrophic lateral sclerosis functional rating scale-revised, SVC=Slow vital capacity, HHD=Hand held dynamometry, ALSAQ-40=Amyotrophic lateral sclerosis assessment questionnaire, VS=Vital sign, BW=Body weight, Ht=Height, PE=Physical examination, ECG=Electrocardiogram, CXR=Chest X-ray, AEs=Adverse events, COM=Concomitant medication, CSF=Cerebrospinal fluid, BME=Bone marrow extraction, MOA=Mode of action, PB=Peripheral blood
